# The Peopling of Europe from the Mitochondrial Haplogroup U5 Perspective

**DOI:** 10.1371/journal.pone.0010285

**Published:** 2010-04-21

**Authors:** Boris Malyarchuk, Miroslava Derenko, Tomasz Grzybowski, Maria Perkova, Urszula Rogalla, Tomas Vanecek, Iosif Tsybovsky

**Affiliations:** 1 Institute of Biological Problems of the North, Far-East Branch of the Russian Academy of Sciences, Magadan, Russian Federation; 2 Department of Molecular and Forensic Genetics, Ludwik Rydygier Collegium Medicum, Institute of Forensic Medicine, The Nicolaus Copernicus University, Bydgoszcz, Poland; 3 Department of Pathology, Medical Faculty Hospital, Charles University, Pilsen, Czech Republic; 4 Centre of Forensic Expertise and Criminalistics, Minsk, Belarus; Natural History Museum of Denmark, Denmark

## Abstract

It is generally accepted that the most ancient European mitochondrial haplogroup, U5, has evolved essentially in Europe. To resolve the phylogeny of this haplogroup, we completely sequenced 113 mitochondrial genomes (79 U5a and 34 U5b) of central and eastern Europeans (Czechs, Slovaks, Poles, Russians and Belorussians), and reconstructed a detailed phylogenetic tree, that incorporates previously published data. Molecular dating suggests that the coalescence time estimate for the U5 is ∼25–30 thousand years (ky), and ∼16–20 and ∼20–24 ky for its subhaplogroups U5a and U5b, respectively. Phylogeographic analysis reveals that expansions of U5 subclusters started earlier in central and southern Europe, than in eastern Europe. In addition, during the Last Glacial Maximum central Europe (probably, the Carpathian Basin) apparently represented the area of intermingling between human flows from refugial zones in the Balkans, the Mediterranean coastline and the Pyrenees. Age estimations amounting for many U5 subclusters in eastern Europeans to ∼15 ky ago and less are consistent with the view that during the Ice Age eastern Europe was an inhospitable place for modern humans.

## Introduction

It has been argued that the most ancient European mitochondrial DNA (mtDNA) haplogroup (hg), U5, arose among the first European settlers in the Upper Paleolithic [1],[2]. Recent molecular dating results suggest that the age of hg U5 oscillates around 36 thousand years (ky), and it has been suggested that any early migration of U5 or its ancestors into Europe might have occurred between ∼55 ky and ∼30 ky ago [3]. There are two U5 subhaplogroups, U5a and U5b, dating back to ∼27 ky each, thus implying that they both originated before the Last Glacial Maximum (LGM) [3]. The frequency of hg U5 in modern European populations is on average 7% [1],[4],[5], but recent studies of ancient mtDNA have shown that U5 haplotypes were common among Mesolithic and Neolithic Europeans, especially of central and eastern parts of Europe [6],[7]. For instance, a high incidence of U5 haplotypes (about 65%) has been detected in European hunter-gatherer individuals [7].

Analysis of mtDNA hypervariable segment I (HVS I) sequences in modern European populations revealed the presence of a bulk of hg U5 subclusters that demonstrated coalescence ages around 11–13 ky and less [8]. Expansions of such U5-subclusters are thought to be linked to favorable climatic changes of the Holocene, allowing re-occupation of large areas of northern Europe by humans from southern European refuges [8]. However, it is unclear whether this process was associated with human dispersals from eastern Europe - partly due to deficiency of the published complete mtDNA sequences from eastern European populations. Therefore, we provide here new information concerning the phylogeny of hg U5 in eastern European populations based on complete mtDNA variability data in Russians, Belorussians, Poles, Czechs and Slovaks, and compare these data with those obtained from western European populations.

## Results

**Figure 1:**
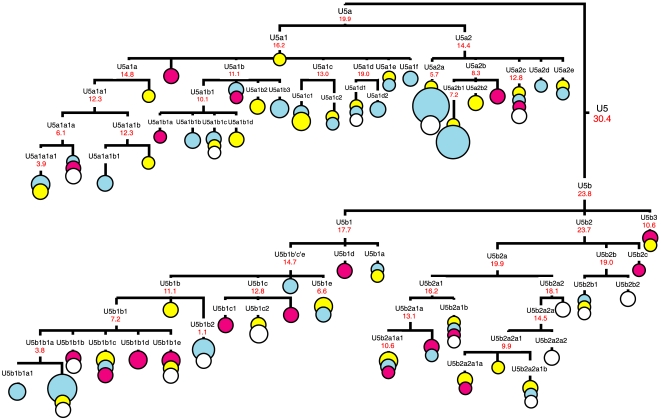
Complete mtDNA phylogenetic tree of haplogroup U5. This schematic tree is based on two phylogenetic trees (for U5a and U5b) presented in Figures S1 and S2, respectively. Nomenclature of hg U5 subclusters was applied in accordance with the classification described in the Table S1. Some unnamed tips are singular haplotypes (*e.g.* U5a1*) aggregated for the purposes of illustration. Time estimates (in ky) shown for mtDNA subclusters are based on the complete mitochondrial genome clock [3]. The size of each circle is proportional to the number of individuals sharing the corresponding haplotype, with the smallest size corresponding to one individual. Geographical origins are indicated by different colors: eastern European – in blue, central European – in yellow, Mediterranean and western European – in fuchsia, and others (*i.e.* of unknown population origin) – in white. More detailed information on the origin of population samples can be found in Figures S1 and S2.

Complete mtDNA genome sequences from eastern European populations allows us to refine the hg U5 phylogeny (Figure 1, Table S1). Subhaplogroup U5a consists of two phylogenetic clusters, U5a1 and U5a2 (Figure S1). Within U5a1, six subclusters can be recognized (from U5a1a to U5a1f). In addition, the founder haplotype for cluster U5a1 has been found in the Czech population (CzIII55). Five subclusters were revealed in cluster U5a2 (from U5a2a to U5a2e). Subhaplogroup U5b is subdivided into three clusters, U5b1, U5b2 and U5b3 (Figure S2). The coalescence time estimate based on the average sequence divergence of the 213 U5 mitochondrial genomes is approximately 25–30 ky (Table 1). Subhaplogroup U5a dates to ∼16–20 ky, implying that it evolved during the LGM, in contrast to subhaplogroup U5b that suggests a pre-LGM time of divergence of 20–24 ky, depending on the mutation rate used. A similar age was found for subhaplogroup U5b2. Coalescence time estimates for several subclusters (U5a1, U5a1a1b, U5a1d, U5a2, U5a2c, U5b1, and U5b2) correspond to the end of the LGM, ∼18 kya, while the remaining U5a and U5b1 subclusters are characterized by lower coalescence ages (Table 1). Therefore, a post-LGM re-expansion of populations from refugial zones between the Pyrenees, the Balkans and the Ukraine, commencing at ∼15 kya, may explain the pattern observed [9].

**Table 1:** Age estimates of hg U5 subclusters calculated using different mutation rates.

Haplogroup	N	Age estimates in ky	
		complete genome rate (95% CI)^a^	synonymous rate (± s.e.)^b^
U5	213	30.4 (21.8; 39.3)	24.8±5.5
U5a	120	19.9 (13.8; 26.1)	16.1±2.6
U5a1	67	16.2 (11.8; 20.7)	14.2±2.5
U5a1a1	21	12.3 (5.4; 19.5)	16.1±6.7
U5a1b	23	11.2 (6.8; 15.7)	9.3±2.3
U5a1c	7	13.0 (6.3; 19.9)	6.8±2.8
U5a1d	7	19.0 (10.5; 27.9)	21.4±8.4
U5a2	53	14.4 (9.1; 19.9)	18.5±5.0
U5a2a	22	5.7 (3.4; 8.0)	9.3±2.6
U5a2b	22	8.3 (6.0; 10.6)	7.5±1.9
U5a2c	5	12.8 (6.6; 19.3)	22.1±7.1
U5b	93	23.8 (17.7; 31.1)	20.2±4.9
U5b1	56	17.7 (9.8; 23.9)	22.8±7.8
U5b1c	5	12.8 (5.9; 20.0)	6.3±3.9
U5b1e	8	6.6 (1.0; 12.3)	5.9±4.2
U5b2	33	23.7 (16.6; 31.2)	16.3±3.6
U5b2a	25	19.9 (13.1; 27.0)	12.9±4.0
U5b2b	6	19.0 (12.1; 26.1)	18.4±5.6
U5b3	4	10.6 (5.0; 16.4)	7.9±3.9

a Mutation rate is one mutation per every 3624 years [3];

b Mutation rate is one mutation per every 7884 years [3].

It has been previously suggested that subcluster U5b1b1 reached northern Europe from an Iberian source, via central/eastern Europe, in the post-LGM times, sometime after ∼15 kya [5],[10]. Tambets et al. [10] have noted the considerable diversity of the U5b1b1 subcluster in western and southern Europe, suggesting that these regions, rather than eastern Europe, were the probable place of origin of U5b1b1. Analysis of complete mitochondrial U5b-genomes indicates that the origin of the whole U5b1 subcluster can be associated with southern and central parts of Europe, because each part of U5b1-phylogeny demonstrates the presence of earlier subclusters of Mediterranean prevalence (such as U5b1b1b, U5b1b1d, U5b1b1e, U5b1c, U5b1d), along with a central European cluster (U5b1a, U5b1b1c, U5b1e) (Figure 1, Figure S2). Among these, subcluster U5b1e is mainly present in central Europe among Czechs, Slovaks, Hungarians and southern Russians (Table S2). The rare subcluster U5b1a has been detected in Mediterranean, southeastern and central European populations, such as Italians, Greeks, Bosnians, Croatians, Slovaks, Hungarians (Table S2).

The most ancient identified subhaplogroup, U5b2, requires further phylogeographic studies. However the data presented here allow us to suggest that at least subcluster U5b2a is characterized by a predominantly central European distribution, since a large number of U5b samples from Poland, Slovakia and the Czech Republic fall into this subcluster (Figure 1). For instance, subcluster U5b2a2 is frequent in central Europe (with the highest frequency of its subcluster U5b2a2a1 in Poles) and dated as arising between 12–18 kya, depending on the mutation rate used (Table 1). It is also remarkable that within U5b2a1a, a Mediterranean branch precedes subcluster U5b2a1a1, which is characteristic of central and eastern European populations (Figure 1). Another U5b subcluster, U5b3, has its most likely homeland in the Italian Peninsula, from where it expanded in the Holocene along the Mediterranean coasts [11]. Hence, in general one may conclude that an initial diversification of U5b occurred in southern and central Europe, followed by the spread of a particular U5b subclusters into eastern Europe.

Subhaplogroup U5a appears to be younger than U5b, and its two subclusters, U5a1 and U5a2, expanded soon after the LGM. Subcluster U5a2 is relatively frequent in central and eastern Europe, but some haplotypes within U5a2b were detected in Mediterranean populations as well (Italians, Tunisians) (Figure 1, Figure S1). Subcluster U5a1 is also present in different populations of central and eastern Europe. It is noteworthy that one of its subclusters, U5a1d, demonstrates a coalescence ages varying from 19 to 21 ky (19.0±4.2 ky for complete mtDNA clock rate to 21.4±8.4 ky for synonymous clock rate), which are comparable with the age of the whole U5a subcluster (Table 1). Subcluster U5a1d has been detected in eastern Europe (in Russians, Belorussians and Tatars) and southern Siberia (in Buryats) (Figure 1, Figure S1). The presence of such old U5a1 lineages in eastern part of Europe may be indicative of eastern European origin of the whole U5a1 subcluster, however ancestral haplotype for U5a1 has been found in central Europe in Czechs (Figure 1).

## Discussion

It has been suggested that hg U5, or a genetically close ancestor to U5, arose among the first settlers of Europe, and subsequently spread all aver the continent along with the Aurignacian industry [1],[2]. According to archaeological data, this Upper Paleolithic industry spread rapidly across western and central Europe roughly 42 to 40 kya, and there is evidence for a slightly earlier influx in southern and central Europe [12]. It is also suggested that Upper Paleolithic occupation at archaeological sites on the Don River in the East European Plain (at Kostenki site) occurred a little earlier, around 45–42 kya [13]. However, taxonomic assignment of paleoanthropological material (teeth) from this site, as well as the relationship between the Kostenki industry and the earliest dated Upper Paleolithic remains in southern and central Europe, remains unclear [13]. A recent study of ancient DNA from the Kostenki site (∼30 kya) in Russia has shown that anatomically modern humans with haplogroup U2 might have populated the East European Plain at that time [14]. Therefore, as in southern and central Europe, only the youngest of the archaeological entities (Aurignacian) in eastern Europe is associated with skeletal remains that may be assigned unequivocally to anatomically modern humans [15],[16].

It is probable, however, that the first successful expansion of modern humans inhabiting the territory of the central part of East European Plain might have occurred at least 24 kya [17]. This follows from the fact that the density of precisely dated sites increased considerably in southern Russia during the Last Glacial Maximum (roughly 24–16 kya), and in north western Russia after degradation of the glacier ice ∼14 kya [18]. Therefore, the hypothesis of the eastern ‘Periglacial refugium’ postulated based on archaeological data [18],[19] is probably supported by genetic data presented here, as the coalescence times for some U5a subclusters (as well as for hg U4 [20]) in eastern Europe correspond to the post-LGM dates. However, we did not detect any mtDNA haplotypes of such antiquity that clearly identify early traces of the pre-LGM expansions in eastern Europe, originating from industries that can be traced back 40–30 ky (for instance, for the Spitsyn or Strelets Cultures assemblages). Evolutionary ages of ∼20–24 ky have been found for ancestors of subhaplogroups U5b and U5b2, which most probably were originated in southern and central part of Europe, because the majority of such haplotypes were revealed in Czechs, Slovaks and Poles and in the Mediterranean populations, based on a set of population data analyzed in the present study (Figure 1, Figure S2).

It is known that the Late Upper Paleolithic industries of central Europe are closely related to contemporaneous eastern European cultures [21],[22]. This probably reflects both the proximity of central Europe, and the presence of similar environments in the Carpathian Basin. The eastern Gravettian industry, which is represented in sites dated back ∼29–22 kya, is found in both central and eastern Europe [22]. However, climates were milder in the Carpathian Basin and, in contrast to the central East European Plain, settlements in central Europe appear to be continuous throughout the LGM [21]. Moreover, the eastern part of central Europe - the Carpathian Basin, is considered by archaeologists and ecologists as one of the European refugia that existed during the LGM [23],[24].

A recent ancient DNA study of Stone Age hunter-gatherers from central and eastern Europe has shown that most of the samples studied (>80%) shared mtDNA haplotypes belonging to haplogroups U5 and U4, haplogroups that notably are relatively rare in central Europe today [7]. Among them, sample 5830a from Hohlenstein-Stadel (Germany), defined as U5a2a, based on the HVS I sequence 16114A-16192-16256-16294-16311, was detected. Its closest HVS I relatives in modern populations can be found among eastern Europeans (in Latvians, Russians, Tatars and Mordvins). In the present study we have completely sequenced two U5a2a mitochondrial genomes with the characteristic transition at np 16311 in Russians (Figure S1). AMS radiocarbon dating of sample 5830a (at ∼7.8 ky old) allows determination of a minimum age for the subcluster U5a2a to which it belongs. Phylogenetic analysis revealed that the coalescent ages of this subcluster vary from 5.7±1.2 ky (for a complete mtDNA clock rate) to 9.3±2.6 ky (for a synonymous clock rate). Therefore, the age of sample 5830a falls within the intervals of these molecular phylogenetic dating. We should note, however, that Russian U5a2a-16311 samples share a mutation at np 9293 with a Belorussian sample, thus forming a subcluster with the age of ∼2.6 ky (Figure S1). Thus it is probable that similar HVS I sequences in Stone Age individuals and modern Russian individuals may be related only within the bounds of the whole subcluster U5a2a, defined, in fact, by parallel mutations at np 16311 originating independently. In addition, another ancient HVS I haplotype, 16192-16270, belonging probably to subcluster U5b1a, was found in skeletons dated to ∼5.6 ky old, from Lithuania [7]. We have found that the age of ancient mtDNAs does not exceed the coalescence time estimates of modern U5b1a sequences, falling within the range of 9.3±3.5 ky (for a complete mtDNA clock rate) to 6.8±4.8 ky (for a synonymous clock rate) (Figure S2). In general, it seems that the combining of phylogenetic and archaeological approaches will be useful for cross-checking data and improvement of human mitochondrial molecular clock estimates, as new information about modern and ancient mitochondrial genomes becomes available [3].

Genetic data obtained in this study allows the suggestion that during the LGM period, central European territories probably represented the area of intermingling between human flow from refugial zones in the Balkans, the Mediterranean coastline and the Pyrenees, as U5a and U5b gene flows occurred from there. Based on dating analysis of the U5 subclusters, it seems very likely that, despite the archaeological evidence testifying to the presence of humans in eastern Europe during the Ice Age, this part of Eurasia might have only been re-populated by modern humans at the end of the LGM, *i.e.* later than central Europe. In addition, U5b gene flow from central to eastern Europe become much more intense after the LGM. In general, we believe that molecular genetic data, in addition to archaeological and fossil evidence, are of significant use for resolving key questions regarding the interaction of human communities and climate.

## Materials and Methods

### Ethics Statement

The study was approved by Bioethics Committee of the Nicolaus Copernicus University in Torun, The Ludwik Rydygier Collegium in Bydoszcz, Poland (statement no. KB/414/2008 from 17 September, 2008). All subjects provided written informed consent for the collection of samples and subsequent analysis.

### Mitochondrial genome sequencing

Out of about 2000 samples that had been screened previously for haplogroup-diagnostic RFLP markers and subjected to control region sequencing [25]–[30], a total of 113 samples representing subclusters U5a (79 samples) and U5b (34 samples) were selected for complete mtDNA sequencing (Table S3). In addition, six U5a samples from South Siberian populations of Buryats and Hamnigans were sequenced to complete the picture of mtDNA variation in northern Eurasia (Table S3). Complete mtDNA sequencing was performed using the methodology described in detail by Torroni et al. [31]. The nucleotide sequences were determined on ABI 3130 Genetic Analyzer using BigDye chemistry (Applied Biosystems, Foster City, CA). DNA sequence data were analyzed using SeqScape v. 2.5 software (Applied Biosystems, Foster City, CA). Mutations were scored relative to the revised Cambridge reference mtDNA sequence [32].

### Phylogenetic analysis

The most-parsimonious trees of the complete mtDNA sequences were reconstructed manually and verified by means of the Network 4.5.1.0 program (www.fluxus-engineering.com), and using mtPhyl program (http://eltsov.org), which is designed to reconstruct maximum parsimony phylogenetic trees. Both programs calculate haplogroup divergence estimates, ρ, and their error ranges as average number of substitutions in mtDNA clusters (haplogroups) from the ancestral sequence type [33]. Values of mutation rates based on mtDNA complete genome variability data (one mutation every 3624 years [3]) and synonymous substitutions (one mutation every 7884 years [3]) were used. To convert ρ values to age estimates with 95% CI bounds we used a calculator provided by Soares et al. [3]. For reconstruction of the hg U5 phylogeny, the data obtained in this study and those published previously by Finnilä et al. [4], Maca-Meyer et al. [34], Palanichamy et al. [35], Achilli et al. [5], Pello et al. [36], Costa et al. [37], Hartmann et al. [38], Pichler et al. [39], as well as FamilyTreeDNA project data available at PhyloTree.org (http://www.phylotree.org) were taken into account. Overall, 213 mitochondrial genomes – 120 U5a and 93 U5b – were analyzed. Nucleotide positions (nps) showing point indels and transversions located between nps 16180–16193 and 303–315 were excluded from the phylogenetic analysis. To evaluate the distribution of subhaplogroups U5a and U5b in Europe and neighboring areas, we performed a survey of the U5 HVS I region motifs reported in almost 17,000 subjects from different population samples (Table S4). The GenBank accession numbers for complete mitochondrial genomes reported in this paper are DQ904330 and GU296541-GU296657.

## Supporting Information

Figure S1 Phylogenetic tree of subhaplogroup U5a, constructed using the program mtPhyl. Numbers along links refer to substitutions scored relative to the revised Cambridge reference mtDNA sequence (rCRS). Transversions are further specified; ins and del denote insertions and deletions of nucleotides, respectively; back mutations are underlined; symbol < denotes parallel mutation. Time estimates based on the complete mitochondrial genome clock [3] (in ky), along with a values of the rho statistic (± sigma) in parentheses, are shown on the branches. Sequences indicated in red print are new (Table S3) while the others have been taken from the literature and accompanied by GenBank numbers and population origin in parentheses. Geographical origins are indicated by different colors: eastern European - in blue, central European - in yellow, Mediterranean and western European - in fuchsia, and others (i.e., of unknown population origin) - in white (unfilled cells).
(0.07 MB XLS)

Figure S2 Phylogenetic tree of subhaplogroup U5b constructed using the program mtPhyl. Numbers along links refer to substitutions scored relative to the rCRS. Transversions are further specified; ins and del denote insertions and deletions of nucleotides, respectively; back mutations are underlined; symbol < denotes parallel mutation. Time estimates based on the complete mitochondrial genome clock [3] (in ky), along with a values of the rho statistic (± sigma) in parentheses, are shown on the branches. Sequences indicated in red print are new (Table S3) while the others have been taken from the literature and accompanied by GenBank numbers and population origin in parentheses. Geographical origins are indicated by different colors: eastern European - in blue, central European - in yellow, Mediterranean and western European - in fuchsia, and others (i.e., of unknown population origin) - in white (unfilled cells).
(0.06 MB XLS)

Table S1 Classification of hg U5 subclusters based on complete mtDNA data.
(0.03 MB DOC)

Table S2 Distribution of mtDNA haplotypes belonging to subclusters U5b1a, U5b1e, U5b2a2a1 in Europe.
(0.08 MB DOC)

Table S3 Control-region variation of the completely sequenced mtDNAs belonging to haplogroup U5.
(0.18 MB DOC)

Table S4 Haplogroup U5a and U5b distribution in different populations of northern Eurasia.
(0.04 MB DOC)
