# Supplementary material for: The Peopling of Europe from the Mitochondrial Haplogroup U5 Perspective
Source: PLoS One. 2010 Apr 21;5(4):e10285. doi: 10.1371/journal.pone.0010285 (PMC2858207; doi:10.1371/journal.pone.0010285)
Supplement: Table S1 — Classification of hg U5 subclusters based on complete mtDNA data. (0.03 MB DOC) [file pone.0010285.s003.doc]

Table S1. Classification of hg U5 subclusters based on complete mtDNA data

_______________________________________________________________

U5: 3197, 9477, 13617, 16270

U5a: 14793, 16192, 16256

U5a1: 15218, 16399

U5a1a: 16192 (back mutation)

U5a1a1: 1700

U5a1a1a: 5495, 15924

U5a1a1a1: 152

U5a1a1b: 12346

U5a1a1b1: 5319, 6629, 6719

U5a1b: 9667

U5a1b1: 16291

U5a1b1a: 4553

U5a1b1b: 8119

U5a1b1c: 9055

U5a1b1d: 16093, 12358

U5a1b2: 9632

U5a1b3: 16362

U5a1c: 16320

U5a1c1: 195, 13802

U5a1c2: 961, 960+C

U5a1d: 3027

U5a1d1: 5263, 13002A

U5a1d2: 3552

U5a1e: 3564, 8610

U5a1f: 6023

U5a2: 16526

U5a2a: 13827, 13928C,16114A, 16294

U5a2b: 9548

U5a2b1: 960+C

U5a2c: 10619

U5a2d: 7843, 7978, 8104, 11107

U5a2e: 151, 152, 3768, 15289, 16189, 16311, 16362

U5b: 150, 7768, 14182

U5b1: 5656

U5b1a: 7028 (back mutation), 15097, 16192

U5b1b'c’e: 16189

U5b1b: 12618

U5b1b1: 7385, 10927

U5b1b1a: 16144

U5b1b1a1: 4059

U5b1b1b: 8413, 16320

U5b1b1c: 16093

U5b1b1d: 15884

U5b1b1e: 16192

U5b1b2: 217, 16192

U5b1c: 15191, 16311

U5b1c1: 14420, 14470

U5b1c2: 516, 16174

U5b1e: 152, 2757, 10283, 12616

U5b1d: 5437, 15721

U5b2: 1721, 13637

U5b2a: 4732

U5b2a1: 16270 (back mutation)

U5b2a1a: 15511

U5b2a1a1: 16311

U5b2a1a1a: 896

U5b2a1b: 152, 5918, 14323, 16189, 16325

U5b2a2: 16189

U5b2a2a: 16192

U5b2a2a1: 3212, 16398

U5b2a2a1a: 9682, 12136

U5b2a2a1b: 2757, 14956

U5b2a2a2: 10031

U5b2b: 11653, 12634, 13630

U5b2b1: 15497

U5b2b2: 4616, 8027

U5b2c: 723, 960+c, 13017

U5b3: 228,7226, 16192, 16304

U5b3a: 16235

U5b3a1: 16169A

U5b3a1a: 373, 11177, 16304 (back mutation)

U5b3a1b: 15781

U5b3a2: 10978

U5b3b: 9196

U5b3c: 4775, 5557, 6461

U5b3d: 13830, 16067, 16311

U5b3e: 3535A, 8701

U5b3f: 16129

_______________________________________________________________

­­­­­­­­­­­­­­
