# Supplementary material for: The Peopling of Europe from the Mitochondrial Haplogroup U5 Perspective
Source: PLoS One. 2010 Apr 21;5(4):e10285. doi: 10.1371/journal.pone.0010285 (PMC2858207; doi:10.1371/journal.pone.0010285)
Supplement: Table S2 — Distribution of mtDNA haplotypes belonging to subclusters U5b1a, U5b1e, U5b2a2a1 in Europe. (0.08 MB DOC) [file pone.0010285.s004.doc]

Table S2. Distribution of mtDNA haplotypes belonging to subclusters U5b1a, U5b1e, U5b2a2a1 in Europe

| Haplogroup | Population | N | Reference |
| --- | --- | --- | --- |
| **U5b1a** |  |  |  |
| 16192-16270 | Bosnians | 1 | Tambets et al. (2004) |
| 16192-16270 | Croatians | 1 | Tambets et al. (2004) |
| 16192-16270 | Italians | 1 | Tambets et al. (2004) |
| 16192-16270 | Latvians | 1 | Tambets et al. (2004) |
| 16192-16270 | Russians | 1 | Present study |
| 16192-16270 | Slovaks | 1 | Malyarchuk et al. 2008 |
| 16192-16270 | Hungarians | 4 | Egyed et al. (2007) |
| 16129-16192-16270 | Swedes | 1 | Tambets et al. (2004) |
| 16192-16270-16296 | Greeks | 1 | Tambets et al. (2004) |
| 16192-16270-16319 | French | 1 | Tambets et al. (2004) |
| 16192-16224-16261-16270 | Hungarians | 1 | Tambets et al. (2004) |
| 16192-16224-16261-16270 | Hungarians | 1 | Egyed et al. (2007) |
| **U5b1e** |  |  |  |
| 16189-16270-16465 | Hungarians | 8 | Egyed et al. (2007) |
| 16189-16270-16465 | Polish | 1 | Present study |
| 16189-16270-16465 | Slovaks | 1 | Present study |
| 16189-16270-16465 | Northern Greeks | 1 | Irwin et al. 2008 |
| 16189-16270-16465-16524 | Czechs | 1 | Present study |
| 16189-16270 | Russians | 1 | Present study |
| 16093-16189-16270-16465 | Russians | 1 | Present study |
| 16093-16189-16270-16465 | Slovaks | 1 | Present study |
| 16093-16189-16270-16465 | Hungarians | 1 | Egyed et al. 2007 |
| 16129-16189-16270-16465 | Slovaks | 1 | Present study |
| **U5b2a2a1** |  |  |  |
| 16189-16270-16398 | Poles | 1 | Malyarchuk et al. 2002 |
| 16189-16270-16398 | Germans | 1 | Lutz et al. 1998 |
| 16189-16270-16398 | Hungarians | 1 | Egyed et al. 2007 |
| 16189-16192-16270-16398 | Belorussians | 1 | Kushnerevich 2008 |
| 16189-16192-16270-16398 | Poles | 2 | Malyarchuk et al. 2002 |
| 16189-16192-16270-16398 | Poles | 2 | Grzybowski et al. 2007 |
| 16189-16192-16270-16398 | Czechs | 1 | Malyarchuk et al 2006 |
| 16189-16192-16270-16398 | Croatians | 1 | GenBank submission |
| 16189-16192-16270-16398 | Transilvanians (Hungaric-speaking) | 1 | Egyed et al. 2007 |
| 16189-16192-16270-16398 | Hungarian Roma | 1 | Egyed et al. 2007 |
| 16189-16192-16270-16398 | Estonians | 1 | Lappalainen et al. 2008 |
| 16189-16192-16270-16398 | Swedes | 1 | Lappalainen et al. 2008 |
| 16189-16192-16270-16398 | Bretons | 1 | Tambets et al. 2004 |
| 16189-16192-16270-16398 | Komi | 1 | Tambets et al. 2004 |
| 16189-16192-16270-16398 | Russian | 1 | Tambets et al. 2004 |
| 16189-16192-16266-16270-16398 | Turks | 1 | Tambets et al. 2004 |
| 16189-16192-16201-16270-16398 | Croatians | 1 | Tambets et al. 2004 |
| 16189-16192-16217-16234-16270-16398 | Tatars | 2 | Tambets et al. 2004 |
| 16189-16217-16234-16270-16398 | Belorussians | 1 | Kushnerevich 2008 |
| 16189-16217-16234-16270-16398 | Poles | 1 | Malyarchuk et al. 2002 |
| 16189-16261-16270-16304-16398 | Slovaks | 1 | Lehocky et al. 2008 |

Note. U5b1a samples from Tambets et al. (2004) study were identified as those characterizing by motif 16192-16270-5656.

**Supplementary Reference List (for population samples analyzed in Tables S2 and S4)**

Achilli A, Olivieri A, Pala M, et al. (2007) Mitochondrial DNA variation of modern Tuscans supports the near eastern origin of Etruscans. Am J Hum Genet 80: 759-768.

Baasner A, Madea B (2000) Sequence polymorphisms of the mitochondrial DNA control region in 100 German Caucasians. J Forensic Sci 45: 1343-1348.

Baasner A, Schafer C, Junge A, Madea B (1998) Polymorphic sites in human mitochondrial DNA control region sequences: population data and maternal inheritance. Forensic Sci Int 98: 169-178.

Bermisheva M, Tambets K, Villems R, Khusnutdinova E (2002) Diversity of mitochondrial DNA haplotypes in ethnic populations of the Volga-Ural region of Russia. Mol Biol (Mosk) 36: 990-1001.

Derbeneva OA, Starikovskaia EB, Volod'ko NV, Wallace DC, Sukernik RI (2002a) Mitochondrial DNA variation in Kets and Nganasans and the early peoples of Northern Eurasia. Genetika 38: 1554-1560.

Derbeneva OA, Starikovskaya EB, Wallace DC, Sukernik RI (2002b) Traces of early Eurasians in the Mansi of northwest Siberia revealed by mitochondrial DNA analysis. Am J Hum Genet 70: 1009-1014.

Derenko M, Malyarchuk B, Grzybowski T, et al. (2007) Phylogeographic analysis of mitochondrial DNA in North Asian populations. Am J Hum Genet 81: 1025-1041.

Egyed B, Brandstätter A, Irwin JA, et al. (2007) [Mitochondrial control region sequence variations in the Hungarian population: analysis of population samples from Hungary and from Transylvania (Romania).](http://www.ncbi.nlm.nih.gov/pubmed/19083748?itool=EntrezSystem2.PEntrez.Pubmed.Pubmed_ResultsPanel.Pubmed_RVDocSum&ordinalpos=19) Forensic Sci Int Genet 1: 158-162.

Grzybowski T, Malyarchuk BA, Derenko MV, et al. (2007) Complex interactions of the Eastern and Western Slavonic populations with other European groups as revealed by mitochondrial DNA analysis. Forensic Sci Int Genet 1: 141-147.

Helgason A, Hickey E, Goodacre S, et al. (2001) mtDNA and the islands of the North Atlantic: estimating the proportions of Norse and Gaelic ancestry. Am J Hum Genet 68: 723-737.

[Irwin J](http://www.ncbi.nlm.nih.gov/sites/entrez?Db=pubmed&Cmd=Search&Term="Irwin J"%5BAuthor%5D&itool=EntrezSystem2.PEntrez.Pubmed.Pubmed_ResultsPanel.Pubmed_RVAbstractPlusDrugs1), [Saunier J](http://www.ncbi.nlm.nih.gov/sites/entrez?Db=pubmed&Cmd=Search&Term="Saunier J"%5BAuthor%5D&itool=EntrezSystem2.PEntrez.Pubmed.Pubmed_ResultsPanel.Pubmed_RVAbstractPlusDrugs1), [Strouss K](http://www.ncbi.nlm.nih.gov/sites/entrez?Db=pubmed&Cmd=Search&Term="Strouss K"%5BAuthor%5D&itool=EntrezSystem2.PEntrez.Pubmed.Pubmed_ResultsPanel.Pubmed_RVAbstractPlusDrugs1), et al. (2008) Mitochondrial control region sequences from northern Greece and Greek Cypriots. Int J Legal Med 122: 87-89.

Kasperaviciute D, Kucinskas V, Stoneking M (2004) Y chromosome and mitochondrial DNA variation in Lithuanians. Ann Hum Genet 68: 438-452.

Kushnerevich EI (2008) Polymorphism of nucleotide sequences of mitochondrial DNA and Y-chromosome of modern native population of Belarus. PhD Thesis. Institute of Genetics and Cytology of National Academy of Belarus Republic, Minsk.

Lappalainen T, Laitinen V, Salmela E, et al. (2008) Migration waves to the Baltic Sea region. Ann Hum Genet 72: 337-348.

Lehocky I, Baldovic M, Kadasi L, Metspalu E (2008) A database of mitochondrial DNA hypervariable regions I and II sequences of individuals from Slovakia. Forensic Sci Int Genet 2: e53-e59.

Lutz S, Weisser HJ, Heizmann J, Pollak S (1998) Location and frequency of polymorphic positions in the mtDNA control region of individuals from Germany. Int J Legal Med 111: 67-77.

Malyarchuk BA, Derenko MV (2001) Mitochondrial DNA variability in Russians and Ukrainians: implication to the origin of the Eastern Slavs. Ann Hum Genet 65: 63-78.

Malyarchuk B, Derenko M, Grzybowski T, et al. (2004) Differentiation of mitochondrial DNA and Y chromosomes in Russian populations. Hum Biol 76: 877-900.

Malyarchuk BA, Grzybowski T, Derenko MV, et al. (2002) Mitochondrial DNA variability in Poles and Russians. Ann Hum Genet 66: 261-283.

Malyarchuk BA, Perkova MA, Derenko MV, et al. (2008) Mitochondrial DNA variability in Slovaks, with application to the Roma origin. Ann Hum Genet 72: 228-240.

Malyarchuk BA, Vanecek T, Perkova MA, et al. (2006) Mitochondrial DNA variability in the Czech population, with application to the ethnic history of Slavs. Hum Biol 78**:** 681-696.

McEvoy B, Richards M, Forster P, Bradley DG (2004) The Longue Duree of genetic ancestry: multiple genetic marker systems and Celtic origins on the Atlantic facade of Europe. Am J Hum Genet 75: 693-702.

Meinilä M, Finnilä S, Majamaa K (2001) Evidence for mtDNA admixture between the Finns and the Saami. Hum Hered 52: 160-170.

Metspalu M, Kivisild T, Metspalu E, et al. (2004) Most of the extant mtDNA boundaries in south and southwest Asia were likely shaped during the initial settlement of Eurasia by anatomically modern humans. BMC Genet 5: 26.

Naumova OY (2008) Mitochondrial DNA diversity in Tobol-Irtysh Siberian Tatars. PhD Thesis. Moscow: Vavilov Institute of General Genetics.

Parson W, Parsons TJ, Scheithauer R, Holland MM (1998) Population data for 101 Austrian Caucasian mitochondrial DNA d-loop sequences: application of mtDNA sequence analysis to a forensic case. Int J Legal Med 111: 124-132.

Pereira L, Cunha C, Amorim A (2004) Predicting sampling saturation of mtDNA haplotypes: an application to an enlarged Portuguese database. Int J Legal Med 118: 132-136.

Pfeiffer H, Brinkmann B, Huhne J, et al. (1999) Expanding the forensic German mitochondrial DNA control region database: genetic diversity as a function of sample size and microgeography. Int J Legal Med 112: 291-298.

Pimenoff VN, Comas D, Palo JU, et al. (2008) Northwest Siberian Khanty and Mansi in the junction of West and East Eurasian gene pools as revealed by uniparental markers. Eur J Hum Genet 16: 1254–1264.

Pliss L, Tambets K, Loogväli EL, et al. (2006) Mitochondrial DNA portrait of Latvians: towards the understanding of the genetic structure of Baltic-speaking populations. Ann Hum Genet 70: 439-458.

Richard C, Pennarun E, Kivisild T, et al. (2007) An mtDNA perspective of French genetic variation. Ann Hum Biol 34: 68-79.

Richards M, Macaulay V, Hickey E, et al. (2000) Tracing European founder lineages in the Near Eastern mtDNA pool. Am J Hum Genet 67: 1251-1276.

Saillard J, Evseeva I, Tranebjaerg L, Norby S (2000) Mitochondrial DNA diversity among Nenets. In: Renfrew C, Boyle K, editors. Archaeogenetics: DNA and the population prehistory of Europe: McDonald Institute for Archaeological Research Monograph Series. Cambridge University Press, Cambridge, p. 255-258.

Sajantila A, Lahermo P, Anttinen T, et al. (1995) Genes and languages in Europe: an analysis of mitochondrial lineages. Genome Res 5: 42-52.

Tambets K, Rootsi S, Kivisild T, et al. (2004) [The western and eastern roots of the Saami – the story of genetic "outliers" told by mitochondrial DNA and Y chromosomes.](http://www.ncbi.nlm.nih.gov/pubmed/15024688?itool=EntrezSystem2.PEntrez.Pubmed.Pubmed_ResultsPanel.Pubmed_RVDocSum&ordinalpos=7) Am J Hum Genet 74: 661-682.

Vanecek T, Vorel F, Sip M. (2004) Mitochondrial DNA D-loop hypervariable regions: Czech population data. Int J Legal Med 118: 14-18.

Zimmermann B, Brandstätter A, Duftner N, et al. (2007) Mitochondrial DNA control region population data from Macedonia. Forensic Sci Int Genet 1: e4-e9.

Zupanič Pajnič I, Balazic J, Komel R (2004) Sequence polymorphism of the mitochondrial DNA control region in the Slovenian population. Int J Legal Med 118: 1-4.
