# Supplementary material for: The Peopling of Europe from the Mitochondrial Haplogroup U5 Perspective
Source: PLoS One. 2010 Apr 21;5(4):e10285. doi: 10.1371/journal.pone.0010285 (PMC2858207; doi:10.1371/journal.pone.0010285)
Supplement: Table S3 — Control-region variation of the completely sequenced mtDNAs belonging to haplogroup U5. (0.18 MB DOC) [file pone.0010285.s005.doc]

Table S3. Control-region variation of the completely sequenced mtDNAs belonging to haplogroup U5

| HVS1 (-16000) | HVS2 | HG | ID | Population |
| --- | --- | --- | --- | --- |
| **U5a** |  |  |  |  |
| 192 256 270 399 | 73 263 309iC 315iC | U5a1 | CzIII55 | Czech |
| 256 270 399 519 | 73 263 309iC 315iC | U5a1a* | Sl43 | Slovak |
| 256 270 399 | 73 152 263 309iC 315iC | U5a1a1a1 | R618 | Russian |
| 256 270 399 | 73 152 263 309iC 315iC | U5a1a1a1 | CzII1 | Czech |
| 256 270 399 | 73 152 263 315iC | U5a1a1a1 | Iv43 | Belorussian |
| 256 261 270 297 399 | 73 152 263 309iC 315iC | U5a1a1a1 | G49 | Polish |
| 256 270 399 | 73 263 315iC | U5a1a1a1 | Iv76 | Belorussian |
| 256 270 399 | 73 152 263 315iC | U5a1a1a1 | Sv22 | Belorussian |
| 256 270 278 399 | 73 152 263 315iC | U5a1a1a1 | R48 | Russian |
| 256 270 399 | 73 152 263 315iC | U5a1a1a1 | R57 | Russian |
| 256 270 399 | 73 152 263 315iC | U5a1a1a1 | R282 | Russian |
| 231 256 270 399 | 73 152 263 315iC | U5a1a1a1 | Ser27 | Polish |
| 256 270 399 | 73 152 263 309iC 315iC | U5a1a1a1 | Bur426 | Buryat |
| 129 256 270 311 399 | 73 146 263 299iC 309iC 315iC | U5a1a1b | B59 | Polish |
| 256 399 | 73 263 315iC | U5a1a1b1 | R856 | Russian |
| 256 399 | 73 263 309iC 315iC | U5a1a1b1 | R752 | Russian |
| 256 270 399 | 73 263 309iCC 315iC | U5a1a1b1 | Bur372 | Buryat |
| 92 192 256 270 291 399 519 | 73 263 315iC | U5a1b1b | R747 | Russian |
| 92 192 209 256 291 399 | 73 263 315iC | U5a1b1b | R449 | Russian |
| 92 192 256 291 399 | 73 263 315iC | U5a1b1b | Sm50 | Belorussian |
| 93 192 256 270 291 399 | 73 263 315iC | U5a1b1b | R594 | Russian |
| 256 270 291 294 399 | 73 263 309iC 315iC | U5a1b1 | B169 | Polish |
| 172 192 256 270 291 311 399 | 73 200 263 315iC | U5a1b1 | Hm22 | Hamnigan |
| 172 256 270 291 399 | 73 263 315iC | U5a1b1c | Ka115 | Polish |
| 51 129 192 256 270 291 399 | 73 249dA 263 315iC | U5a1b1c | R6212 | Russian |
| 192 256 270 291 399 | 73 263 315iC | U5a1b1c | R472 | Russian |
| 93 192 256 291 399 | 73 263 315iC | U5a1b1d | B388 | Polish |
| 93 192 256 270 291 399 | 73 198 263 315iC | U5a1b1d | G129 | Polish |
| 192 256 270 291 399 519 | 73 195 198 263 315iC | U5a1b | R736 | Russian |
| 192 256 270 291 399 519 | 73 195 198 263 315iC | U5a1b | R405 | Russian |
| 176 192 256 270 278 399 | 73 263 315iC | U5a1b2 | CzIII17 | Czech |
| 192 256 270 399 | 73 263 315iC | U5a1b2 | CzI24 | Czech |
| 192 256 270 362 399 428 | 73 263 315iC | U5a1b3 | R5 | Russian |
| 192 256 270 286 320 399 | 73 183 263 315iC | U5a1c2 | B93 | Polish |
| 192 256 302 320 390 399 | 73 199 263 309iC 315iC | U5a1c2 | R578 | Russian |
| 86 192 256 270 320 399 | 73 195 263 309iC 315iC | U5a1c1 | R738 | Russian |
| 192 256 270 320 399 | 73 195 207 263 315iC | U5a1c1 | CzV78 | Czech |
| 256 270 320 399 | 73 195 263 309iC 315iC | U5a1c1 | Sl25 | Slovak |
| 192 256 270 320 399 | 73 195 263 309iC 315iC | U5a1c1 | R427 | Russian |
| 192 256 270 320 399 | 73 195 263 309iC 315iC | U5a1c1 | P553 | Polish |
| 51 192 256 270 399 | 73 263 309iC 315iC | U5a1d1 | B36 | Polish |
| 93 172 192 256 270 399 | 73 241 263 309iC 315iC | U5a1d1 | R479 | Russian |
| 145 189 192 256 270 399 | 73 195 263 309iC 315iC | U5a1d2 | Sv102 | Belorussian |
| 92 145 189 192 256 270 399 | 73 195 263 309iC 315iC | U5a1d2 | R171 | Russian |
| 145 189 192 256 270 399 | 73 195 263 309iC 315iC | U5a1d2 | Bur340 | Buryat |
| 192 256 270 399 | 73 152 263 315iC | U5a1e | B95 | Polish |
| 129 192 256 270 399 | 73 263 315iC | U5a1e | R1109 | Russian |
| 192 222 256 270 399 | 73 203 204 263 309iCC 315iC | U5a1f | R354 | Russian |
| 114A 192 256 270 294 311 526 | 73 263 309iC 315iC | U5a2a | R131 | Russian |
| 114A 192 256 270 294 311 526 | 73 263 309iC 315iC | U5a2a | R533 | Russian |
| 114A 192 256 270 294 526 | 73 263 309iC 315iC | U5a2a | Sm59 | Belorussian |
| 114A 192 256 270 294 526 | 73 263 309iC 315iC | U5a2a | R425 | Russian |
| 114A 192 256 270 294 526 | 73 263 309iC 315iC | U5a2a | Iv31 | Belorussian |
| 114A 192 256 270 294 526 | 73 263 309iC 315iC | U5a2a | R7411 | Russian |
| 114A 192 256 270 294 526 | 73 263 309iC 315iC | U5a2a | B128 | Polish |
| 114A 167 192 256 270 294 526 | 73 263 309iC 315iC | U5a2a | G80 | Polish |
| 114A 192 256 270 294 526 | 73 263 309iC 315iC | U5a2a | R730 | Russian |
| 114A 192 256 270 294 526 | 73 263 315iC | U5a2a | R2121 | Russian |
| 114A 256 270 294 526 | 73 263 315iC | U5a2a | R6240 | Russian |
| 114A 192 256 270 294 526 | 73 263 315iC | U5a2a | R111 | Russian |
| 114A 192 256 270 294 526 | 73 263 309iC 315iC | U5a2a | R324 | Russian |
| 192 256 270 311 526 | 73 263 315iC | U5a2b1 | R740 | Russian |
| 192 256 270 526 | 73 263 315iC | U5a2b1 | B167 | Polish |
| 192 256 270 526 | 73 263 315iC | U5a2b1 | R6237 | Russian |
| 192 256 270 526 | 73 263 315iC | U5a2b1 | R857 | Russian |
| 189 192 256 270 526 | 73 263 315iC | U5a2b1 | R6238 | Russian |
| 93 192 256 270 526 | 73 211 263 315iC | U5a2b1 | Sm77 | Belorussian |
| 192 256 270 526 | 73 263 315iC | U5a2b1 | R431 | Russian |
| 192 256 270 526 | 73 263 315iC | U5a2b1 | Iv10 | Belorussian |
| 136 192 256 270 526 | 73 263 315iC | U5a2b1 | R761 | Russian |
| 192 256 270 526 | 73 263 315iC | U5a2b1 | Sv105 | Belorussian |
| 192 256 270 526 | 73 263 315iC | U5a2b1 | Sv24 | Belorussian |
| 192 256 270 526 | 73 263 315iC | U5a2b1 | R20 | Russian |
| 147G 256 270 311 465 526 | 73 263 315iC | U5a2b1 | CzI14 | Czech |
| 192 218 256 270 278 465 526 | 73 263 315iC | U5a2b1 | R444 | Russian |
| 192 256 269 270 526 | 73 150 263 315iC | U5a2b1 | R178 | Russian |
| 192 256 270 311 526 | 73 263 315iC | U5a2b1 | R6221 | Russian |
| 192 234 256 270 491 526 | 73 263 309iC 315iC | U5a2b2 | P34 | Polish |
| 192 234 256 270 526 | 73 263 309iC 315iC | U5a2b2 | Sl198 | Slovak |
| 192 256 270 | 73 263 315iC | U5a2b | Bur634 | Buryat |
| 192 256 270 | 73 263 315iC | U5a2b | Hm55 | Hamnigan |
| 256 526 | 73 263 293 315iC | U5a2c | Ser43 | Polish |
| 192 256 270 526 | 73 263 315iC | U5a2c | R476 | Russian |
| 189 192 256 270 311 362 526 | 73 151 152 263 309iC 315iC | U5a2e | CzI16 | Czech |
| 189 192 234 256 270 311 362 526 | 73 151 152 263 315iC | U5a2e | Sm3 | Belorussian |
| **U5b** |  |  |  |  |
| 192 270 | 73 150 204 207 235 263  315iC | U5b1a | R628 | Russian |
| 129 144 189 270 | 73 150 263 315iC | U5b1b1a | Sv36 | Belorussian |
| 144 189 270 | 73 150 263 309iCC 315iC | U5b1b1a | Sl102 | Slovak |
| 144 189 270 | 73 150 263 309iC 315iC | U5b1b1a | Iv26 | Belorussian |
| 144 189 270 | 73 150 263 309iC 315iC | U5b1b1a | R137 | Russian |
| 93 129 189 270 | 73 150 263 315iC | U5b1b1c | R879 | Russian |
| 93 189 270 | 73 150 263 315iC 384 | U5b1b1c | CzII5 | Czech |
| 93 189 270 | 73 150 263 309iC 315iC | U5b1b1c | B341 | Polish |
| 189 192 270 | 73 150 263 315iC | U5b1b1e | CzII16 | Czech |
| 147 183C 189 270 | 73 150 263 309iCC 315iC | U5b1b* | Sl123 | Slovak |
| 140 174 183C 189 193iC 270 288 311 | 73 150 263 309iC 315iC | U5b1c2 | B384 | Polish |
| 182C 183C 189 270 519 | 73 150 263 309iC 315iC | U5b1b’c | Iv45 | Belorussian |
| 93 183C 189 270 465 | 73 150 152 263 315iC | U5b1e | Sl30 | Slovak |
| 189 270 465 | 73 150 152 263 315iC | U5b1e | Sl128 | Slovak |
| 129 189 270 465 | 73 150 152 263 285 309iC 315iC | U5b1e | Sl239 | Slovak |
| 189 270 | 73 150 152 263 315iC | U5b1e | R6225 | Russian |
| 93 183C 189 193iC 270 465 | 73 150 152 263 315iC | U5b1e | R6232 | Russian |
| 189 270 465 524 | 73 150 152 263 315iC | U5b1e | CzIV18 | Czech |
| 189 270 465 | 73 150 152 263 315iC | U5b1e | Ks414 | Polish |
| 16519 | 73 150 263 309iC 315iC | U5b2a1a | R755 | Russian |
| 174 192 311 | 73 150 263 315iC | U5b2a1a | Sl91 | Slovak |
| 192 311 | 73 150 263 309iC 315iC | U5b2a1a | CzIII5 | Czech |
| 86 239 311 320 | 73 150 263 315iC | U5b2a1a | B72 | Polish |
| 189 264 325 | 73 150 152 263 315iC | U5b2a1b | R457 | Russian |
| 189 325 | 73 150 152 263 315iC | U5b2a1b | B85 | Polish |
| 189 192 270 398 | 73 150 263 315iC | U5b2a2a1b | Sv108 | Belorussian |
| 189 192 270 398 | 73 150 263 315iC | U5b2a2a1b | CzII21 | Czech |
| 189 192 270 398 | 73 150 263 315iC | U5b2a2a1b | P4 | Polish |
| 189 217 234 270 398 | 73 150 263 315iC | U5b2a2a1 | B2 | Polish |
| 189 192 270 398 | 73 150 263 315iC | U5b2a2a1a | B328 | Polish |
| 189 270 398 | 73 150 263 315iC | U5b2a2a1a | B248 | Polish |
| 234 240C 270 362 519 | 73 150 263 309iC 315iC | U5b2b1 | R2132 | Russian |
| 270 526 | 73 150 263 315iC | U5b2b1 | Sl61 | Slovak |
| 270 304 | 73 150 228 263 315iC | U5b3 | CzI44 | Czech |

Note. Variants are transitions (with respect to the revised CRS), transversions are further specified. “i” and “d” is insertion and deletion of the indicated nucleotide(s), respectively.
