# Supplementary material for: The Peopling of Europe from the Mitochondrial Haplogroup U5 Perspective
Source: PLoS One. 2010 Apr 21;5(4):e10285. doi: 10.1371/journal.pone.0010285 (PMC2858207; doi:10.1371/journal.pone.0010285)
Supplement: Table S4 — Haplogroup U5a and U5b distribution in different populations of northern Eurasia. (0.04 MB DOC) [file pone.0010285.s006.doc]

Table S4. Haplogroup U5a and U5b distribution in different populations of northern Eurasia

| Regions | Populations | U5a | U5b |
| --- | --- | --- | --- |
| Mediterranean region (n=3431) | Greeks, Italians, Portuguese, Spaniards | 3.1 (105) | 2.3 (78) |
| southeastern Europe (n=812) | Bulgarians, Romanians, Bosnians, Slovenians, Macedonians | 6.4 (52) | 3.6 (29) |
| north-central Europe (n=2566) | Hungarians, Austrians, Germans, Poles, Czechs, Slovaks | 6.0 (155) | 3.8 (98) |
| Scandinavia (n=863) | Swedes, Norwegians | 6.4 (55) | 5.9 (51) |
| northwestern Europe (n=2297) | French, Irish, Scottish, Wales | 4.8 (111) | 3.6 (82) |
| eastern Europe (n=3730) | Russians, Ukrainians, Belorussians, Mordva, Maris, Chuvash, Finns, Karelians, Estonians, Latvians, Lithuanians, Nenets, Udmurts, Komi, Tatars | 7.7 (288) | 5.8 (218) |
| the Ural and western Siberia (n=487) | Bashkirs, Nganasans, Khants, Mansis, Kets | 6.4 (31) | 2.3 (11) |
| the Caucasus (n=1999) | different ethnic groups | 6.3 (125) | 1.1 (22) |
| Iran (n=518) | different locations | 3.3 (17) | 0.6 (3) |

Note. Number of individuals are shown in parentheses. References for population data are as in the Table S2.
